# Reverse transcriptase activity and particles of retroviral density in cultured canine lymphosarcoma supernatants.

**DOI:** 10.1038/bjc.1983.36

**Published:** 1983-02

**Authors:** F. M. Tomley, S. J. Armstrong, B. W. Mahy, L. N. Owen

## Abstract

Lymphoid tissue from 43 cases of canine lymphosarcoma and from 40 clinically normal dogs have been examined for markers of retrovirus infection. From 69-76% of culture supernatants from lymphosarcomas were shown to contain particles of retroviral density and to possess poly rC-oligo dG templated polymerase (reverse transcriptase) activity compared with 17-24% of culture supernatants from normal canine lymphoid cells. In 6 culture supernatants from cases of lymphosarcoma, high molecular weight 60-70S RNA was detected and shown to be found in association with this particulate reverse transcriptase activity. No such RNA was detected in 6 culture supernatants from normal canine lymphoid cells.


Br. J. Cancer (1983), 47, 277–284

### Reverse transcriptase activity and particles of retroviral density in cultured canine lymphosarcoma supernatants

F. M. Tomley, S. J. Armstrong, B.W.J. Mahy* & L. N. Owen

*Department of Clinical Veterinary Medicine, University of Cambridge, & *Division of Virology, Department of Pathology, University of Cambridge, Cambridge, U.K.*

**Summary** Lymphoid tissue from 43 cases of canine lymphosarcoma and from 40 clinically normal dogs have been examined for markers of retrovirus infection. From 69–76% of culture supernatants from lymphosarcomas were shown to contain particles of retroviral density and to possess poly rC–oligo dG templated polymerase (reverse transcriptase) activity compared with 17–24% of culture supernatants from normal canine lymphoid cells. In 6 culture supernatants from cases of lymphosarcoma, high molecular weight 60–70S RNA was detected and shown to be found in association with this particulate reverse transcriptase activity. No such RNA was detected in 6 culture supernatants from normal canine lymphoid cells.

Lymphosarcoma is one of the most common malignancies found in the dog, with a reported incidence of 20 per $10^5$ adult dogs (Jarrett *et al.*, 1966). The tumours can be transplanted by intrafoetal inoculation of unrelated canines with tumour cells (Owen & Neilson, 1968), the malignancies becoming apparent in the puppies about one month after birth. Cell-free transmission of lymphosarcoma has never been successful, but the cell-free transmission of canine mast-cell leukaemia has been reported (Rickard & Post, 1968).

Retroviruses are the known aetiological agents of lymphoid neoplasia in several domestic species, including cats, chickens and cattle (for review, see Klein, 1980). Recent evidence strongly indicates that a retrovirus is the causative agent of certain types of cutaneous T cell leukaemia and lymphoma in man (Poiesz *et al.*, 1981; Yoshida *et al.*, 1982). In dogs, there have been reports of electron microscopic observation of retrovirus-like particles in low density in some tumours (Seman *et al.*, 1967; Rudolph *et al.*, 1971) but another study failed to observe such particles (Rangan *et al.*, 1971). Onions (1980) reported finding Reverse Transcriptase activity in the supernatants of 3/14 short-term lymphosarcoma cultures and in 2/11 crude preparations of tumour tissue. Finally, the isolation of a retrovirus from a canine lymphoma B-cell line has been reported (Strandstrom & Rimaila-Parnanon, 1979 and in personal communication).

We have undertaken to survey tissue from both lymphosarcomatous canines and clinically normal controls for evidence of retroviral activity, using 2 simple *in vitro* screening assays, *viz.* assay for reverse transcriptase (RT) activity and detection of particles of retroviral density using sucrose gradient ultracentrifugation.

Correspondence: F.M. Tomley
Received 14 October 1982; accepted 14 November 1982.

### Materials and methods

### *Sources of material*

Forty-three cases of lymphosarcoma (WHO Classification), referred to the Oncology Unit for therapy between October 1980 and April 1982, were examined in this survey. Where possible, a lymph node biopsy was taken prior to therapy for histological diagnosis and for tissue culture. In 23 cases this was not possible, either because of the poor state of the animal or because a biopsy for histological purposes had already been performed by the referring veterinary surgeon. Where possible, bodies were obtained for *post-mortem* and further tissues (bone marrow, lymph nodes, spleen and heparinised blood) taken for tissue culture and biochemical examination.

Forty-one clinically normal dogs were also examined. These were obtained as fresh cadavers from local veterinary surgeons.

It proved impossible to match breeds of lymphosarcoma cases with breeds of normal dogs. Twenty-one different breeds with lymphosarcoma were examined, with cross-breeds, Labradors and German Shepherd dogs predominating. Control dogs consisted predominantly of Greyhounds no longer economic to race and cross-breeds, Labradors, Border Collies, Beagles and others euthanased for a variety of reasons such as savageness, old age, accident etc. Upon histological examination none of the control animals was found to show evidence of neoplasm. Because of limitations on time and finance, only lymph nodes were were cultured from control animals.

0007-0920/83/020277-08 $02.00 © The Macmillan Press Ltd., 1983

*Culture of primary material*

Solid tissues were chopped into 2 mm pieces and pressed through a grade 50 mesh. Samples of bone marrow and buffy coat cells (from heparinised peripheral blood) were resuspended in Hanks basal salts solution (HBSS). All cell preparations were washed in HBSS, layered onto 5.8% Ficoll–8.5% Hypaque gradients, centrifuged at 2,000 g for 15 min, and the interface cells removed and washed once more. These cells were then counted and resuspended at $5 \times 10^5$ ml$^{-1}$ in RPMI 1640 medium (Gibco) supplemented with 15% foetal calf serum, 2 mM L-glutamine, and 0.75 $\mu$g ml$^{-1}$ Concanavalin A.

*Assay for reverse transcriptase activity*

Primary cultures were incubated for 18–24 h and the culture supernatants clarified by centrifugation at 10,000 g for 30 min. The resultant supernatant was pelleted by centrifugation at 75,000 g for 90 min and pellets resuspended in 300 $\mu$l (per original 30 ml of supernatant) of disruption buffer which contained 50 mM Tris-HCl, pH 8.0, 0.04% Nonidet P40, and 20 mM dithiothreitol (DTT). The lysates were kept on ice for 30 min prior to assay, then duplicate volumes of sample (50 $\mu$l) were mixed with an equal volume of assay mixture to give 50 mM Tris-HCl pH 8.0, 10 mM DTT, 50 mM NaCl, 1.0 mM $MnCl_2$, or 10 mM $MgCl_2$, 1 unit ml$^{-1}$ 20:1 poly (rC)–oligo $(dG)_{12-18}$ and 20 $\mu$Ci ml$^{-1}$ [$^3$H]-dGTP (10 Ci mM$^{-1}$). The mixtures were incubated at 37°C. Similar incubations were carried out using a DNA template-primer, (1 unit ml$^{-1}$ 20:1 poly (dA)–oligo $(dT)_{12-18}$) in place of poly rC–oligo dG, with 20 $\mu$Ci ml$^{-1}$ [$^3$H]-dTTP (10 Ci mmol$^{-1}$) as the labelled nucleoside precursor. This provided a measure of DNA dependent–DNA polymerase activity in the samples. The acid-insoluble radioactivity after 60 min incubation was determined and termed $T_{60}$, and a control $T_0$ value was determined by measuring the incorporation achieved by duplicate samples which were acid-precipitated at zero time. Avian myeloblastosis virus (AMV) Reverse Transcriptase was used as a positive control and PBSA as a negative control.

*Assay of [$^3$H]-Uridine labelled material in culture supernatants*

Primary cultures were incubated for 18–24 h in the presence of 20 $\mu$Ci ml$^{-1}$ of 5.6 [$^3$H]-uridine, 48 Ci mM$^{-1}$ (Amersham International Ltd). Culture supernatants (30 ml) were clarified and pelleted as above and pellets resuspended in 300 $\mu$l of NTE buffer (100 mM NaCl, 10 mM Tris-HCl pH 7.8, 1 mM EDTA). Resuspended pellets were layered on top of preformed 4.5 ml 20–60% w/v sucrose gradients which were centrifuged for 18 h at 80,000 g then fractionated from the bottom into ~25 fractions. Fifty-$\mu$l was removed from each, the refractive index read, and the density determined. A further 100 $\mu$l was removed into assay tubes and the acid-insoluble radioactivity measured.

*Sizing of RNA and simultaneous detection assays*

Primary cultures were incubated for 18–24 h in the presence of 20 $\mu$Ci ml$^{-1}$ 5.6 [$^3$H]-uridine, and the supernatants clarified and pelleted as above. Pellets were resuspended in NTE buffer, made 1% with sodium dodecyl sulphate and extracted by shaking at 25°C for 5 min with an equal volume of phenol:cresol (7:1 pH 7.6) containing 37 mg ml$^{-1}$ 8-hydroxyquinolene. After centrifugation at 5,000 g for 5 min, the aqueous phase was layered over a preformed 4.5 ml 10–30% v/v glycerol gradient and centrifuged at 100,000 g for 3 h. Gradients were fractionated, the density of each fraction determined and the acid-insoluble radioactivity measured. The size of peaks was estimated by using labelled RNA markers of known S value.

Primary cultures were incubated for 18–24 h and the supernatants clarified and pelleted as above. Pellets were resuspended in 300 $\mu$l of disruption buffer (50 mM Tris HCl pH 8.0, 0.04% NP40, 20 mM DTT), and the simultaneous assay of reverse transcriptase activity directed by a high molecular weight template was performed as described by Schlom & Spiegelman (1971).

*Determination of acid-insoluble radioactivity*

To each assay tube, on ice, 80 $\mu$g of native calf thymus DNA then 0.4 ml of 20% Trichloracetic acid (TCA) in 0.125 M sodium pyrophosphate were added and incubated on ice for 20 min. The acid insoluble precipitate was collected onto Whatman 2.4 mm GF/C filters which were washed 3 × with cold 5% TCA and once with cold absolute ethanol. Filters were air dried overnight, placed in scintillation vials with 4 ml of a toluene-based scintillation cocktail (Fisofluor, Fisons) and the acid-insoluble c.p.m. counted in a Packard Tricarb liquid scintillation counter.

### Results

*Age, sex and treatment distributions*

The mean age of the 43 lymphosarcomatous canines examined was 6 yrs 5 months with a spread from 6 months to 12y. Fifty-eight percent were male, 42% female.

Table I outlines the spread of cases between those which were treated with either chemotherapy (vincristine sulphate, cyclophosphamide and prednisolone), radiotherapy (half body X-irradiation), both or neither, and indicates the number of cases where samples were obtained either before or after treatment. In 15 cases, a single lymph node biopsy was obtained before the onset of therapy. In a further 3 cases, this initial biopsy was followed up by *post-mortem* examination. In 23 cases, *post-mortem* examination alone was available, and these can be split into 12 untreated cases, 5 cases which had received chemotherapy, 2 which had received radiotherapy and 4 which had received both. The majority of tissues examined were from animals which received no treatment. In only 14/43 cases had any therapy been instigated. It is regrettable that in only 3 cases were we able to look at lymph node biopsies prior to treatment in addition to examining cadavers at post-mortem.

The mean age of 41 clinically normal canines examined was 5 y 4 months with a spread from 9 months to 13 y. Fifty-five percent were male, 45% female. In all cases, lymph nodes only were removed at *post-mortem.*

**Table I** Distribution of age, sex and therapy in cases of lymphosarcoma examined in the survey

| | *No. Males* | *No. Females* | *Total Number* | *Mean Age (y)* |
|---|---|---|---|---|
| Biopsy | 7 | 8 | 15 | 6.8 |
| Biopsy and Post Mortem. | 3 | 0 | 3 | 7.7. |
| Post Mortem—(untreated) | 7 | 5 | 12 | 5.0 |
| Post-Mortem—(chemotherapy) | 3 | 2 | 5 | 8.3 |
| Post-Mortem—(radiotherapy | 1 | 1 | 2 | 8.5 |
| Post-Mortem—(combined therapy) | 2 | 2 | 4 | 7.5 |
| Other[a] | 2 | 0 | 2 | 5.6 |

[a]One case white blood cells only, the other white blood cells and bone marrow aspirate.

*Classification of lymphosarcoma cases*

The anatomical type of 40 cases seen was multicentric with 1 case alimentary and 2 cases of lymphocytic leukaemia. Histologically, lymphosarcoma in dogs has been classified according to the WHO system: (1) poorly differentiated (2) lymphoblastic (3) lymphocytic and pro-lymphocytic, and (4) histiocytic, histioblastic and histiolymphocytic.

Of the multicentric cases, 28 were lymphoblastic, 5 pro-lymphocytic, 4 histiocytic and 3 poorly differentiated.

Tumours were not classified into B or T cell origin.

*Reverse transcriptase (R.T) assays*

R.T. assays were performed on 72 samples from lymphosarcomatous dogs and on 40 lymph node samples from control dogs. Within the lymphosarcoma group, 17 of these samples were lymph node biopsies and the remainder were samples of bone marrow, spleen, thymus, lymph node or white blood cells, taken at *post-mortem.*

An overall comparison of R.T. activity directed by poly (rC)–oligo (dG) in lymphosarcomatous tissue and in normal canine lymph nodes is shown in Figure 1. Using scattergrams to show the frequency of distribution of $T_{60}$–$T_0$ values for both groups, it can be seen that the values fall into 2 clusters, and by taking the natural cut-off point of 800 c.p.m., the % of tissues regarded as positive for R.T. activity can be calculated for both groups. Seventy-six percent of lymphosarcomatous tissue was R.T. positive compared to 17% of control lymph nodes. A more valid comparison between lymphosarcomas and controls is to look solely at lymph node cultures since only these were cultured for the controls. Sixty-nine percent of lymph nodes from lymphosarcomas were found to be R.T. positive. One point of great interest is that, when looking at cases where more than one tissue type was tested, only one case (*LS81-21*) out of 20 had all tissues negative by this R.T. assay, i.e. 95% of lymphosarcomas have at least one tissue positive.

None of the culture supernatants, either from lymphosarcomatous or normal dogs, were found to produce significant levels of polymerase activity using poly dA–oligo dT as template-primer. This confirms that the activity is dependent on an RNA template primer, and eliminates the possibility that the observed incorporation might result, in part, from a DNA terminal transferase activity.

The poly rC–oligo dG directed activity functions in the presence of either $Mn^{2+}$ or $Mg^{2+}$, but as shown in Table II, the highest incorporation is achieved using 1 mM $MnCl_2$. Using $MgCl_2$ the maximal incorporation achieved is less than two thirds of that achieved using $MnCl_2$. The activity prefers NaCl at 40–50 mM and functions at a greatly reduced efficiency if KCl is substituted.

*Detection of particles of retroviral density*

Detection of particles by [$^3$H]-uridine labelling, followed by sucrose gradient ultracentrifugation

**Figure 1** The distribution of poly rC–oligo dG templated reverse transcriptase (R.T.) activity in cases of A. lymphosarcoma and B. clinically normal dogs. Assays were carried out as described in the text and values expressed as $\log_{10}T_{60}-T_0$. Each point represents the mean of triplicate assays performed on a particular culture supernatant. The cut-off line at 2.9 (800 c.p.m.) represents an arbitrary division of positive and negative samples.

was performed on 69 lymphosarcomatous tissues and on 25 lymph node samples from control dogs. Of the lymphosarcomatous tissues, 14 were lymph node biopsies and the remainder were samples taken at post-mortem.

Figure 2 shows the gradient profile from a post-mortem culture of lymph node tissue from case *LS81-6*. There is a clear high peak of incorporation at $1.16\,g\,ml^{-1}$ which is in the expected range of densities for retroviruses. However, in many cases, the gradient profiles are not so striking, and in order to give an overall comparsion of profiles from lymphosarcomatous and normal tissues, the values were processed as follows. For each gradient profile, the mean c.p.m. per fraction was calculated

**Table II** Divalent cation (A) and salt preferences (B) of poly rC–oligo dG templated reverse transcriptase activity in high speed pellet from canine lymphosarcoma culture supernatant

| *Divalent Cation* | *R.T. Activity (c.p.m.)* | *Salt* | *R.T. Activity (c.p.m.)* |
|---|---|---|---|
| A. 0.5 mM $MnCl_2$ | 8,107 | B. 20 mM NaCl | 6,149 |
| 1.0 mM $MnCl_2$ | 12,179 | 45 mM NaCl | 12,891 |
| 1.5 mM $MnCl_2$ | 5,672 | 80 mM NaCl | 11,770 |
| 5 mM $MgCl_2$ | 6,547 | 20 mM KCl | 7,666 |
| 10 mM $MgCl_2$ | 7,547 | 45 mM KCl | 7,547 |
| 15 mM $MgCl_2$ | 6,056 | 80 mM KCl | 3,296 |

Lymph node cells from case *LS82-3* were seeded at $5\times10^5\,ml^{-1}$ and the supernatant harvested at 24 h; 30 ml of this supernatant was pelleted and resuspended as described in the text and each assay was performed using 50 $\mu$l of the resuspended material.

Results are expressed as c.p.m. of [$^3$H] dTMP incorporated into product after 60 min incubation. The range of concentrations shown in the Table are those which gave the highest incorporation levels in the assay.

**Figure 2** A sucrose gradient centrifugation of [$^3$H]-uridine labelled high-speed pellet from lymph node cell supernatant of case *LS81-6*. The pellet was centrifuged through a 20–60% (w/v) sucrose gradient for 18 h at 80,000 g as described in the text, and acid precipitable radioactivity and density of each fraction determined.

both for the density range of retroviruses (1.14–1.19 g ml$^{-1}$) and for the remainder of the profile, and a ratio of the 2 values, denoted peak/background, calculated. Thus, the case shown in Figure 2 has a ratio of 4.3. These values were then plotted as scattergrams to show the frequency distribution of peak/background ratios for both lymphosarcomatous and normal tissue. Figure 3 shows these scattergrams. The points do not separate into 2 clusters as markedly as in the R.T. activity scattergrams, but, nevertheless, there is clearly a bigger proportion of lymphosarcomatous tissues with high peak/background ratios compared to the control group. If a cut-off point of 1.5 is taken, then 74% of the lymphosarcomatous tissue is positive and 24% of control lymph nodes are positive. Taking solely lymph node cultures, then 72% of the lymphosarcomas are positive.

**Figure 3** The distribution of [$^3$H]-uridine labelled peaks of retroviral density detected on sucrose gradients, in cases of lymphosarcoma A and clinically normal dogs B. Values are expressed as $\log_{10}$ peak/background as explained in the text. The cut-off line at 0.17 (peak/background ratio of 1.5) represents an arbitrary division of positive and negative samples.

Again, if cases with more than one tissue type are examined, only 1/20 has all its tissues negative for particles of retroviral density, and this was the same case (*LS81-21*) found to be completely negative by R.T. assay.

It was found that if [$^3$H]-uridine labelled pellets were incubated at 0°C in disruption buffer for 20 min prior to layering on gradients, a shift in density of the particles occurred from 1.15–1.17 to 1.20–1.23 g ml$^{-1}$. This also occurred if samples were left in the refrigerator for 48 h in NTE buffer. In addition, in 34% of samples which had peaks at 1.15–1.17 g ml$^{-1}$ there was a secondary peak in the 1.20–1.23 region of the gradient.

Table III summarises the results of the survey.

Not all tissues were assayed for both R.T. activity and particles of retroviral density. Of the 63 that were, 51 (81%) showed agreement between the 2 tests and 12 (19%) showed disagreement.

**Table III** Summary of survey

| A. *Reverse Transcriptase* | *No. tested* | *No. positive* | *No. negative* |
|---|---|---|---|
| All lymphosarcoma tissue. | 72 | 55(76%) | 17(24%) |
| Lymphosarcomas—lymph nodes only. | 39 | 27(69%) | 12(31%) |
| Control lymph nodes. | 40 | 7(17%) | 33(83%) |
| B. [$^3$*H*]-*uridine* | *No. tested* | *No. positive* | *No. negative* |
| All lymphosarcoma tissue. | 69 | 51(74%) | 18(26%) |
| Lymphosarcomas—lymph nodes only. | 36 | 26(72%) | 10(28%) |
| Control lymph nodes. | 25 | 6(24%) | 19(76%) |

### *70S RNA and simultaneous detection assays*

These assays were performed on 6 lymph node samples from cases which were highly positive for both R.T. ($T_{60}-T_0 > 2{,}500$ c.p.m.) and [$^3$H]-uridine (peak/background > 4). In all 6 samples a peak of RNA was found at the 60-70S position, and, in 3 of the samples, an additional peak at 35S was also seen. Both peaks could be abolished by pretreatment of the sample with 50 $\mu$g ml$^{-1}$ RNAse A. Figure 4 shows the profile of RNA for case *LS81-6*.

In the simultaneous detection assays, again peaks of incorporation were found at 60–70S position,

**Figure 4** The detection of 70S RNA in a [$^3$H]-uridine labelled high speed pellet from a lymph node culture supernatant of case *LS81-6*. The RNA was isolated from the pellet as described in the text and centrifuged through a 10–30% (v/v) glycerol gradient for 3 h at 100,000 g.

**Figure 5** The simultaneus detection of [$^3$H] DNA complexed to 60–70S and 35S RNA in high speed pellet from a lymph node culture supernatant of case *LS81-6*.

and in all but one case, additional peaks at 35S were observed. Figure 5 shows the RNA–cDNA profile for case *LS81-6*.

Six lymph node samples from control animals which were negative by R.T. and [$^3$H]-uridine assays were also tested by both these assays. No peaks in the 70 or 35S regions were observed.

### Discussion

Examination of lymphoid tissue from 43 lymphosarcomatous and 40 clinically normal dogs has shown the presence of an RNA dependent DNA polymerase activity, and particles of 1.15–1.18 g ml$^{-1}$, in both populations. In the lymphosarcoma culture supernatants, from 69–76% of the tissues examined show significant levels of poly rC–oligo dG templated polymerase activity compared to 17–24% of control lymph node cultures. The data need careful consideration since R.T. assay results can be open to misinterpretation. However, the lack of significant activity with poly dA–oligo dT implies that the results are not due to DNA dependent DNA polymerases or terminal transferases. In some of the cases examined, R.T. levels were raised only a few hundred c.p.m. above background but in 37% of cases the activity was several thousand c.p.m. Similarly with the [$^3$H]-uridine assays, the variation in peak/background ratios was marked from case to case, and in 34% of cases there was an additional peak of incorporation at 1.20–1.23 g ml$^{-1}$, which is the known density of retroviral core particles. Since a consistent volume (30 ml) of supernatant from cultures seeded at $5 \times 10^5$ ml$^{-1}$, was always assayed, then these variations in levels of detectable activity presumably reflect some variation either in the activity itself, or in the state of the tissues examined. Apart from differences in age and sex, which do not appear to have any significant effect on the level of activity detected, the other major considerations are the type of therapy, if any, that the animal had received and the anatomical and histological classification of the disease.

Untreated animals, either at biopsy or at *post-mortem,* certainly have fewer R.T. positive tissues than those examined at *post-mortem* after therapy. However, whether this is specifically because therapy, particularly radiotherapy, predisposes the tissues to expression of R.T. activity, due to some immunosuppressive effect, or whether it is because therapy prolongs the course of the disease, therefore allowing greater expression, is not clear. Of the 3 cases which were examined both at biopsy, then after therapy at *post-mortem,* only one showed any differences in R.T. and [$^3$H]-uridine assays. This dog was negative by both tests upon initial examination of lymph node tissue, but 4 weeks later following half body irradiation, all tissues examined were strongly positive. Further studies into the effects of therapy on R.T. activity are continuing, and of particular interest is the effect of chemotherapy and radiotherapy on the R.T. status of clinically normal dogs.

There was no correlation between the anatomical or histological classification of the disease and the presence or absence of retroviral markers, with cases of each type being positive in one or more tissue.

The variation in levels of R.T. activity observed here is not unique. In particular, the relatively low $T_{60}-T_0$ values (<1,500 c.p.m.) found in 73% of the R.T. positive cases is consistent with the findings of Onions (1980) who detected similar levels of R.T. activity in canine lymphosarcoma cultures. Low but detectable levels of R.T. activity have similarly been reported in other species, for example rabbits (Bedigian *et al.*, 1976) and baboons (Benveniste *et al.*, 1974) and in the latter case infectious virus was eventually isolated once a suitable host was found. Work is currently in progress to investigate the infectivity of this putative canine virus both *in vitro* and *in vivo.*

In 6 of the highly positive cases, the observed particles have been shown to contain 60–70S RNA which is found in association with the poly rC–oligo dG templated polymerase activity. This lends additional support to the premise that there is a retrovirus associated with these cases. The 60–70S RNA appears to be readily degraded into 35S subunits, particularly when subjected to simultaneous detection assay which involves more preparation stages than simple determination of RNA size. Degradation of retroviral RNA into 35S subunits after denaturation is well documented (Duesberg, 1968) and reflects the characteristic dimeric structure of retroviral nucleic acids (for review see Chien *et al.*, 1980).

In conclusion, the survey presented here of both lymphosarcomatous and clinically normal canines, indicates that there is, associated with both groups, a particulate enzyme activity which will incorporate deoxynucleoside triphosphates into an acid-insoluble product in the presence of a DNA-primed RNA template. The activity is associated with particles which have a buoyant density of 1.16–1.18 $g\,ml^{-1}$ in sucrose and which possess a high molecular weight RNA in association with the polymerase. Whether these observations are due to the presence of a canine retrovirus, found particularly in association with lymphosarcoma, and whether there is any aetiological relevance remains to be determined.

This work was supported by a grant from the Leukaemia Research Fund. We would like to thank Mrs. A Martin and Mrs. J. Hale for skilled technical assistance and Mrs. J. Tickner for the typing of this manuscript.

**References**

BEDIGIAN, H.G., FOX, R.R. & MEIER, H. (1976). Presence of a high-molecular weight DNA and RNA-directed polymerase in rabbit hereditary lymphosarcoma. *Cancer Res.* **36,** 4693.

BENVENISTE, R.E., LIEBER, M.M., LIVINGSTÒNE, D.M., SHEIR, C.J., TODARO, G.J. & KALTER, S.S. (1974). Infectious C-type virus isolated from a baboon placenta. *Nature* **248,** 17.

CHIEN, Y. JUNGHANS, R.P. & DAVIDSON, N. (1980). Electron microscopic analysis of the structure of RNA tumor virus nucleic acids. In *Molecular Biology of DNA Tumor Viruses,* J.R. Stephenson (Ed) Academic Press Inc. p. 396.

DUESBERG, P.H. (1968). Physical properties of Rous sarcoma virus RNA. *Proc. Nat Acad. Sci.,* **60,** 1511.

JARRETT, W.F., CRIGHTON, G.W. & DALTON, R.G. (1966). Leukaemia and lymphosarcoma in animals and man. *Vet. Rec.,* **79,** 693.

KLEIN, G. (1980). In *Viral Oncology.* New York: Raven Press.

ONIONS, D. (1980). RNA dependent DNA polymerase activity in canine lymphosarcoma. *Eur. J. Cancer,* **16,** 345.

OWEN, L.N. & NEILSON, S.W. (1968). Transplantation of canine lymphosarcoma. *Eur. J. Cqncer,* **4,** 391.

POIESZ, B.J., RUSCETTI, R.W., REITZ, M.S., KALYANARAMAN, V.S. & GALLO, R.C. (1981). Isolation of a new type C retrovirus (HTLV) in primary uncultured cells of a patient with Sézary T-cell leukemia. *Nature,* **294,** 268.

RANGAN, S.R.S., CALVERT, R.C. & VITOL, S.K. (1971). Fibrillar bundles in canine lymphomas. An ultrastructural study. *J. Ultrastruct. Res.,* **36,** 425.

RICKARD, G.G. & POST, J.E. (1968). Cellular and cell-free transmission of a canine mast cell leukaemia. *In Bendixen Leukaemia in Animals and Man.* Basel: Karger. p. 279.

RUDOLPH, R. (1971). Virsähnliche strukturen in Tumourzellen bei lymphatischer Leukose des Hundes. *Berl. Münch. Tierärztl. Wscher.*, **84**, 68.

SCHLOM, J. & SPIEGELMAN, S. (1971). Simultaneous detection of reverse transcriptase and high molecular weight RNA unique to Oncogenic RNA viruses. *Science*, **174**, 840.

SEMAN, G., PROENCA, G., GIULLON, J.C. & MORAILLON, R. (1967). Partic d'aspect viral dans les cellules du lymphosarcoma du chien. *Bull. Acad. Vet.* (*France*) **4**, 211.

STRANDSTROM, H.V. & RIMAILA-PARNANEM, E. (1979). Canine atypical malignant lymphoma. *Am. J. Vet. Res.*, **40**, 1033.

YOSHIDA, M., MIYOSHI, I. & HINUMA, Y. (1982). Isolation and characterisation of retrovirus from cell lines of human adult T-cell leukaemic and its implication in the disease. *Proc. Nat Acad. Sci.*, **79**, 2031.


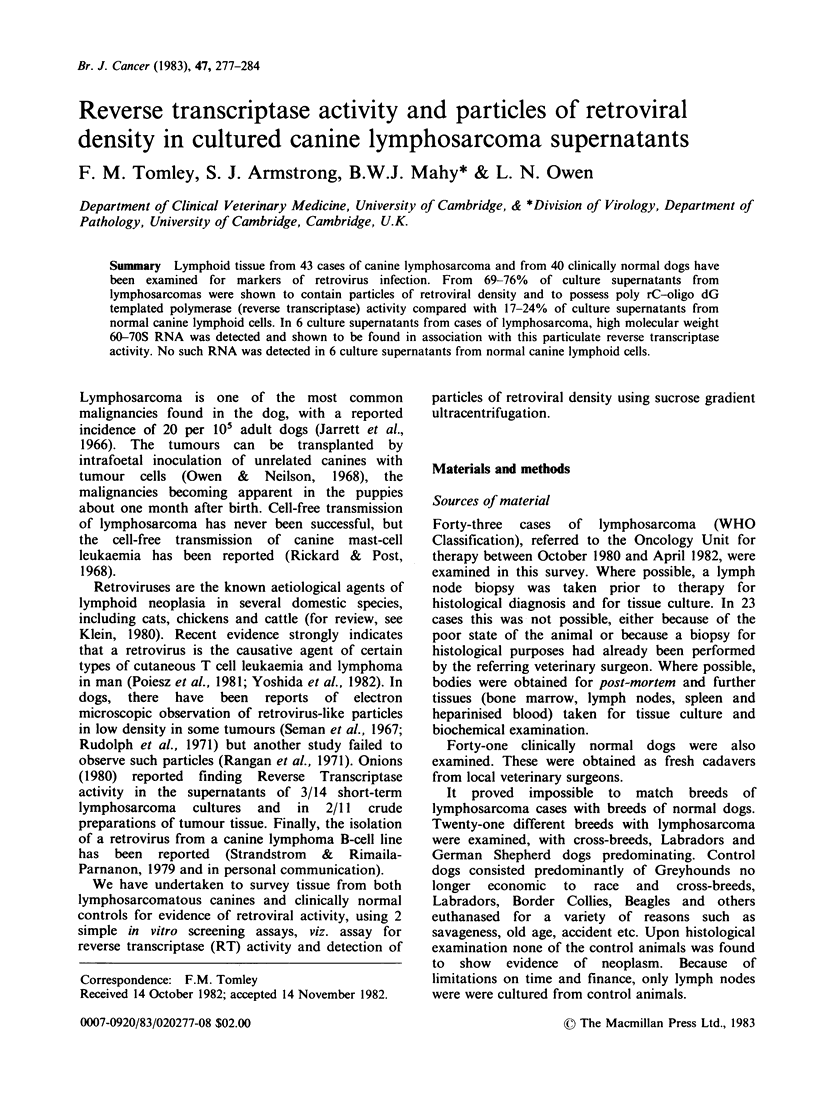

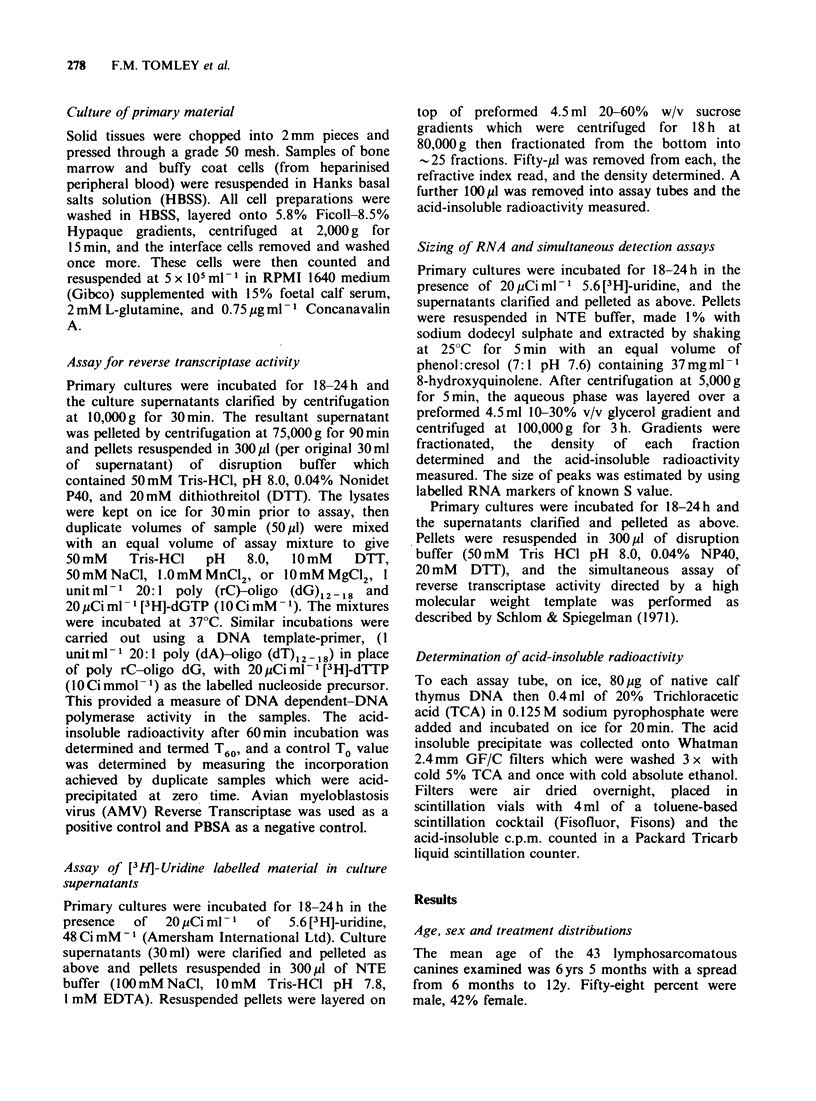

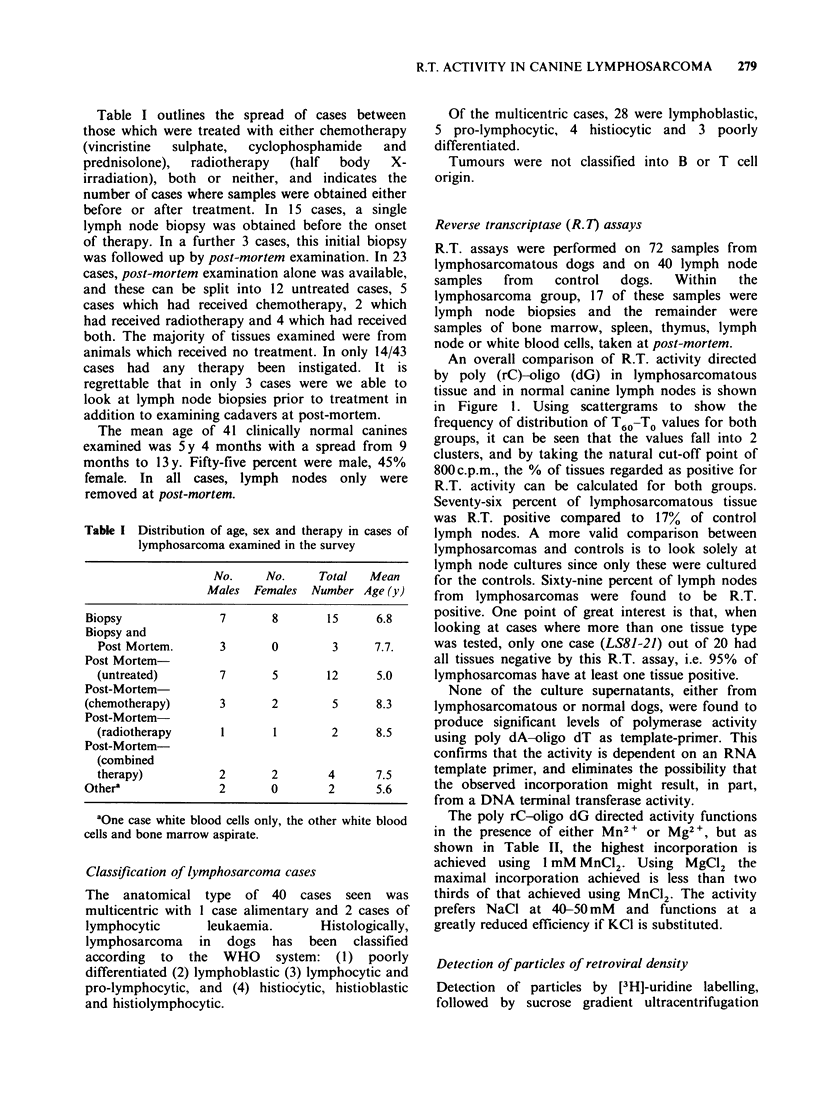

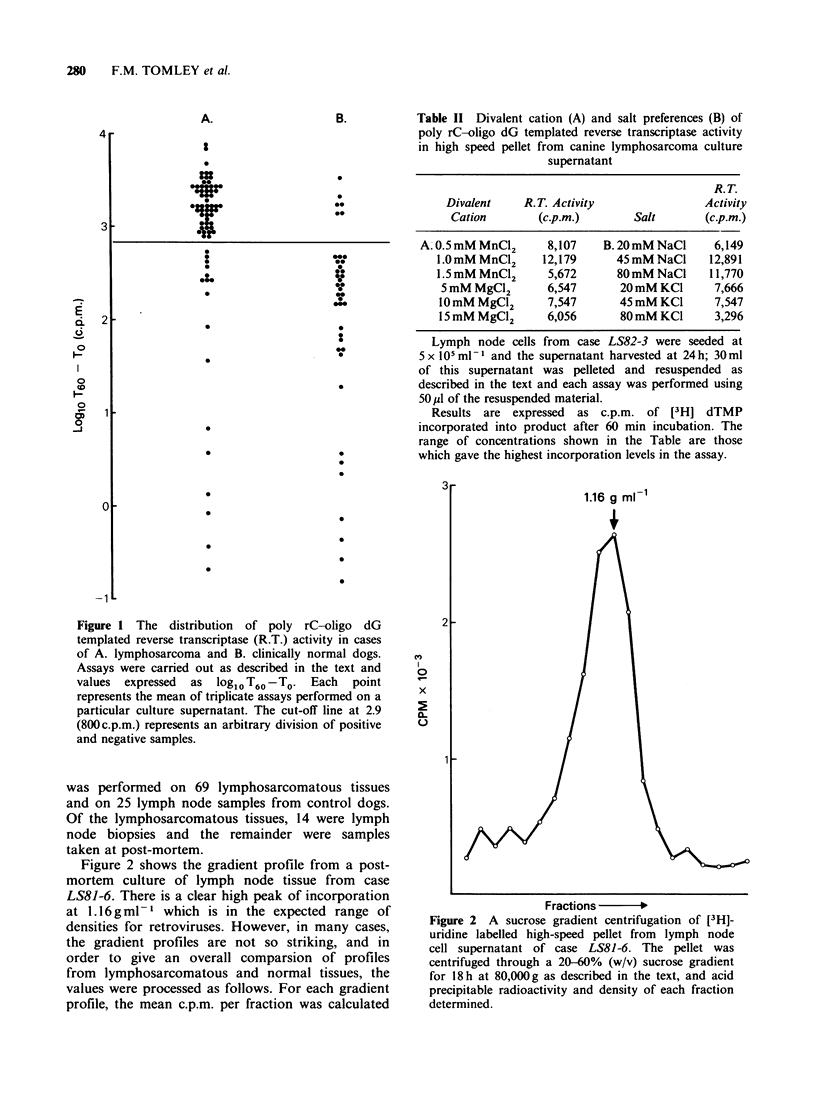

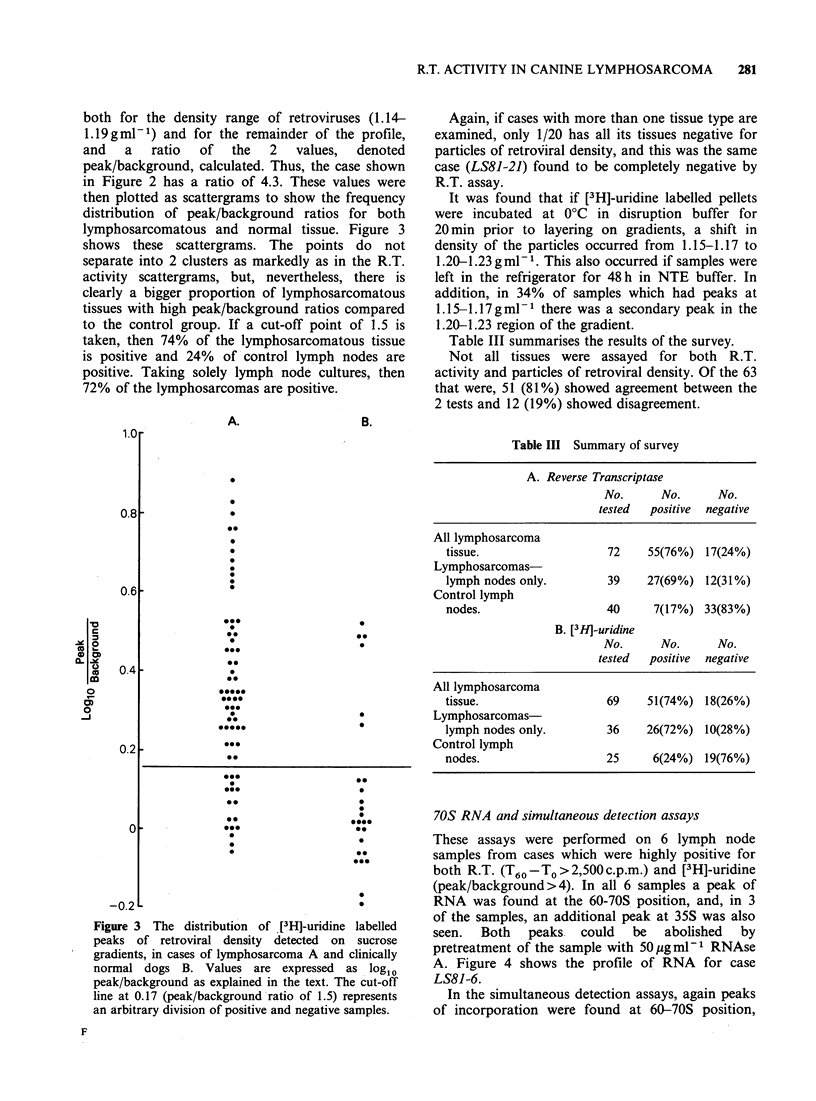

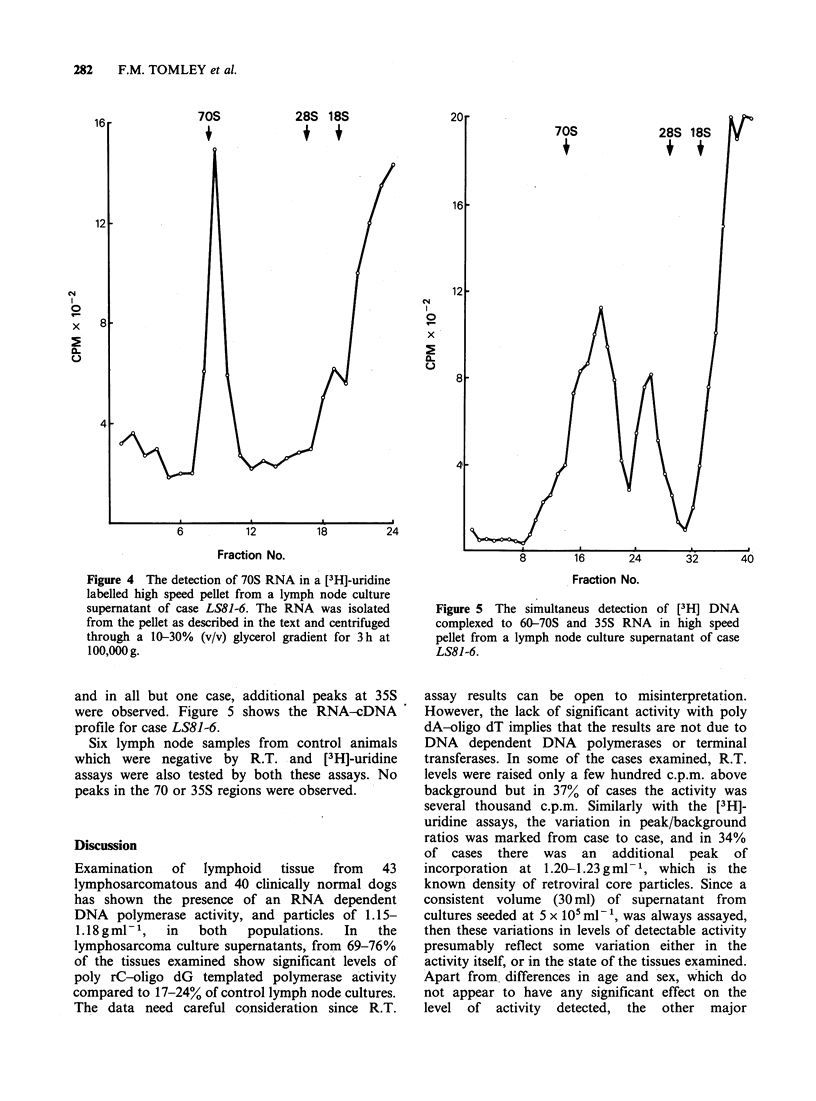

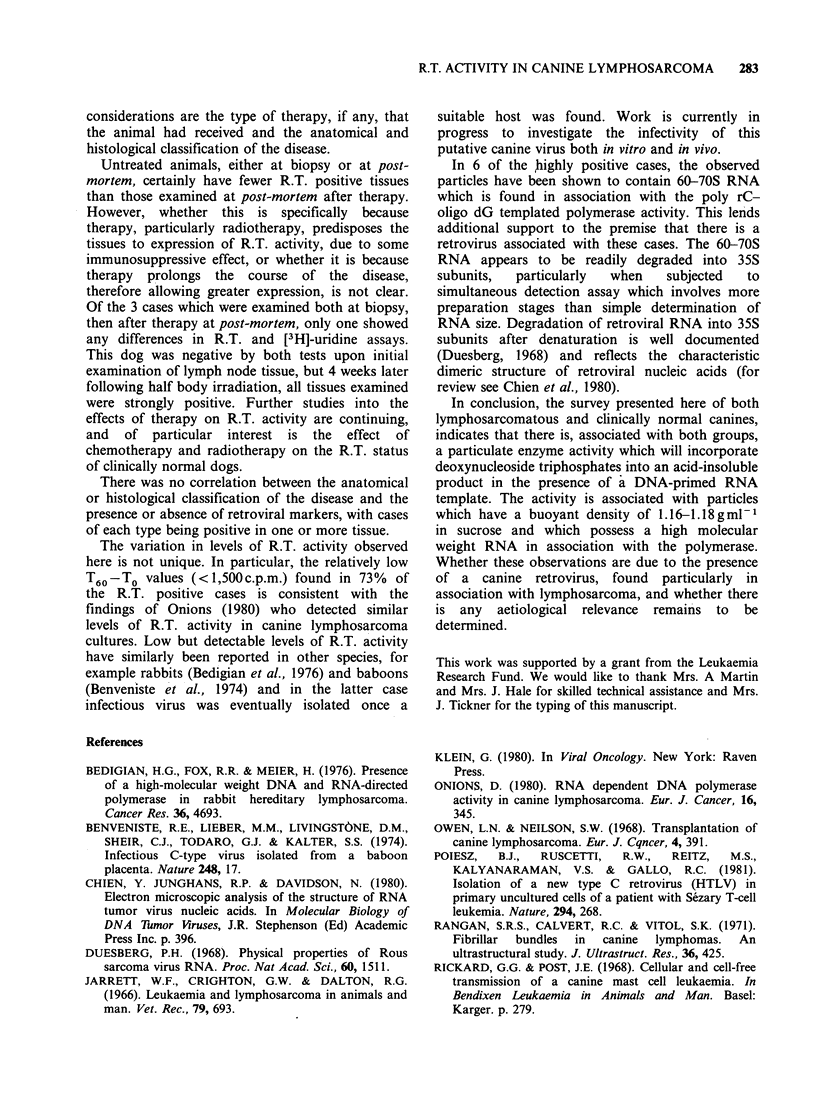

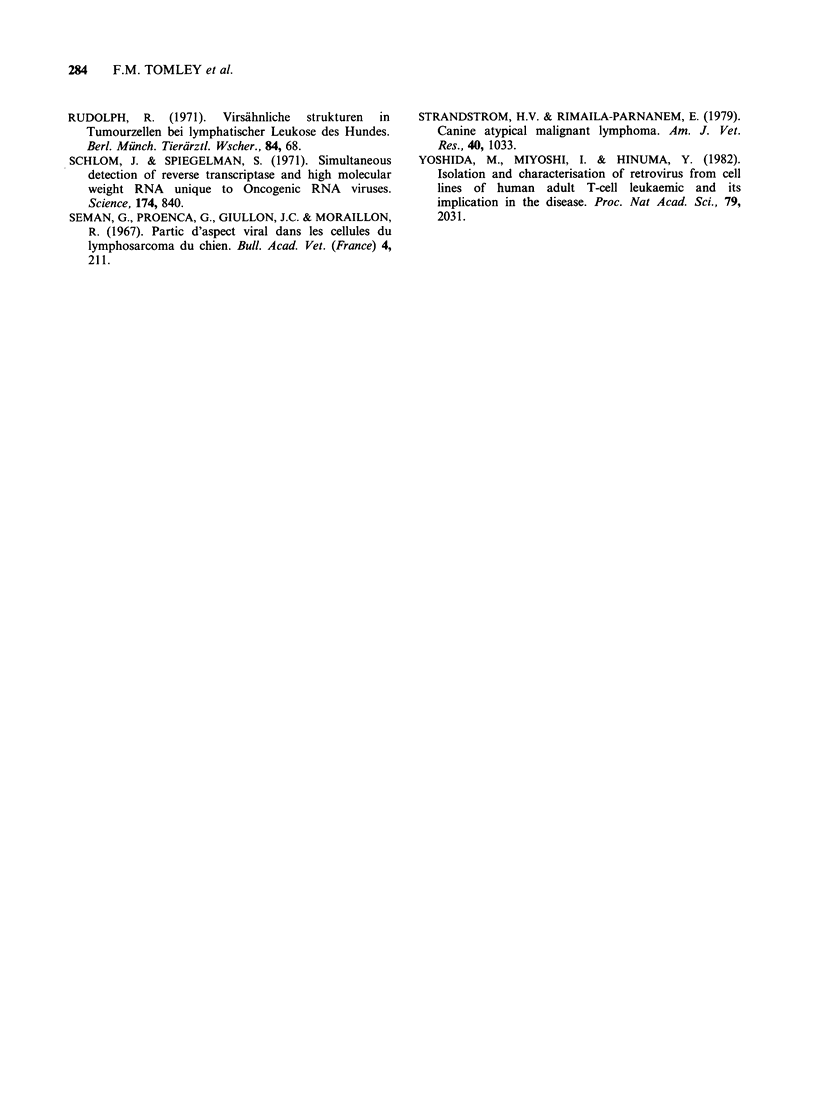


## References

[OCR_00854] Bedigian H. G., Fox R. R., Meier H. (1976). Presence of a high-molecular-weight RNA and RNA-directed DNA polymerase in rabbit hereditary lymphosarcoma.. Cancer Res.

[OCR_00860] Benveniste R. E., Lieber M. M., Livingston D. M., Sherr C. J., Todaro G. J., Kalter S. S. (1974). Infectious C-type virus isolated from a baboon placenta.. Nature.

[OCR_00873] Duesberg P. H. (1968). Physical properties of Rous Sarcoma Virus RNA.. Proc Natl Acad Sci U S A.

[OCR_00886] Onions D. (1980). RNA-dependent DNA polymerase activity in canine lymphosarcoma.. Eur J Cancer.

[OCR_00891] Owen L. N., Nielsen S. W. (1968). Transplantation of canine lymphosarcoma.. Eur J Cancer.

[OCR_00895] Poiesz B. J., Ruscetti F. W., Reitz M. S., Kalyanaraman V. S., Gallo R. C. (1981). Isolation of a new type C retrovirus (HTLV) in primary uncultured cells of a patient with Sézary T-cell leukaemia.. Nature.

[OCR_00902] Rangan S. R., Calvert R. C., Vitols K. (1971). Fibrillar bundles in canine lymphomas: an ultrastructural study.. J Ultrastruct Res.

[OCR_00915] Rudolph R. (1971). Virusähnliche Strukturen in Tumorzellen bei lymphatischer Leukose des Hundes.. Berl Munch Tierarztl Wochenschr.

[OCR_00920] Schlom J., Spiegelman S. (1971). Simultaneous detection of reverse transcriptase and high molecular weight RNA unique to oncogenic RNA viruses.. Science.

[OCR_00926] Seman G., Proenca G., Guillon J. C., Moraillon R. (1967). Particles d'aspect viral dans les cellules du lymphosarcome du chien.. Bull Acad Vet Fr.

[OCR_00932] Strandstrom H. V., Rimaila-Parnanen E. (1979). Canine atypical malignant lymphoma.. Am J Vet Res.

[OCR_00937] Yoshida M., Miyoshi I., Hinuma Y. (1982). Isolation and characterization of retrovirus from cell lines of human adult T-cell leukemia and its implication in the disease.. Proc Natl Acad Sci U S A.

